# Baseline C-Reactive Protein Levels and Life Prognosis in Parkinson Disease

**DOI:** 10.1371/journal.pone.0134118

**Published:** 2015-07-28

**Authors:** Hideyuki Sawada, Tomoko Oeda, Atsushi Umemura, Satoshi Tomita, Masayuki Kohsaka, Kwiyoung Park, Kenji Yamamoto, Hiroshi Sugiyama

**Affiliations:** Department of Neurology and Clinical Research Center, National Hospital of Utano, National Hospital Organization, Kyoto, Japan; Chiba University Center for Forensic Mental Health, JAPAN

## Abstract

**Background:**

C-reactive protein (CRP) is a biomarker of inflammation, and high levels of CRP correlate with vascular death. Chronic inflammation is considered to be involved in neurodegeneration, although there is no evidence linking it with the process of neurodegenerative diseases.

**Objective:**

To determine the role of baseline CRP levels in the prognosis of patients with Parkinson disease (PD).

**Methods:**

A cohort of 313 patients with a mean age of 69.1 and mean PD duration of 7.9 years was retrospectively followed for a mean observation time of 1,753 days. CRP was measured when patients were not diagnosed with any infections, and levels were repetitively measured to investigate a tendency of “regression to mean.” The primary outcome measure was a survival time from study enrollment to death.

**Results:**

During the observation period 56 patients died. Baseline CRP was log-linearly associated with a risk of death in PD. Mean survival time was 3,149 (95% confidence interval; 3,009-3,289) days in patients with CRP ≤ 0.8mg/L (lower two thirds) and 2,620 (2,343-2,897) days in those with CRP > 0.8 mg/L (top third, *p* < 0.001, log-rank test). The adjusted hazard ratio (HR) per two-fold higher CRP concentration for all deaths was 1.29 (1.10-1.52), and after excluding PD-unrelated deaths, such as cancer or stroke, HR was 1.23 (1.01-1.49) (adjusted for age, sex, PD duration, modified Hohen-Yahr stages, MMSE scores, and serum albumin).

**Conclusions:**

Baseline CRP concentrations were associated with the risk of death and predicted life prognosis of patients with PD. The associations were independent from PD duration, PD severity, cognitive function, ages, and nutritional conditions, suggesting the possibility that subclinical chronic inflammation is associated with a neurodegenerative process in PD.

## Introduction

The level of C-reactive protein (CRP) in the peripheral blood is used as a biomarker of systemic inflammation, and is associated with increased risk of coronary heart disease [1], vascular death [2], and cancer death [3]. Baseline CRP levels (except for acute inflammation) are stable, similar to those of blood pressure or serum cholesterol [4].

While chronic inflammation is thought to be associated with neurodegeneration [5, 6], its relation to the prognosis of patients with brain diseases remains uncertain. Previous studies have reported a causal relationship between systemic inflammation and functional changes in the brain, such as “sickness behavior” [5, 7], as well as microglia activation in affected brain areas in Alzheimer disease [1], together with the generation of CRP in these brain lesions [8].

Parkinson disease (PD) is a neurodegenerative disorder characterized pathologically by dopaminergic neuronal death and the presence of Lewy bodies. Although the exact cause of the disease remains to be identified, neuroinflammatory mechanisms may contribute to the neurodegenerative disease process [9]. Pathological studies have demonstrated microglial activation in autopsied PD brains [10, 11], and this finding has been confirmed in neuroimaging studies using PK-11159, a marker of microglia activation, especially in the early stages of PD [12]. CRP is synthesized in hepatocytes, and plasma CRP concentrations are transiently and significantly elevated during acute inflammation [13]. In contrast, CRP concentrations are stable during the non-inflammatory phase [4]. A molecular and cellular communication between peripheral inflammation and the brain have been proposed [14]. In this context, it is reasonable to assume that CRP levels at baseline, i.e., in non-inflammatory conditions, are stable, and that such levels influence long-term prognosis by modulating the neurodegenerative process.

Although functional prognosis in PD is typically evaluated using indexes related to various clinical features, such as motor function, motor complications, autonomic failure, and cognitive decline, these indexes could be subjective and biased. Life prognosis is a solid endpoint with a definite inter-rater reliability and is, therefore, a suitable endpoint in retrospective analysis. In this study, we focused on life prognosis instead of functional prognosis to investigate the relationship between CRP and PD prognosis. We hypothesized that baseline CRP levels are associated with the neurodegenerative process. To test this hypothesis, we conducted a retrospective cohort study and determined the association between baseline plasma CRP levels and PD life prognosis.

## Methods

### Study design

For the purpose of the study, a retrospective cohort of PD patients was followed to determine the relationship between baseline CRP concentrations and PD life prognosis. First, we compared survival time of patients according to sex (male *vs*. female), age (young *vs*. elderly), PD disease duration (short *vs*. long), PD severity (modified Hoehn-Yahr stage), nutritional condition (high serum albumin *vs*. low serum albumin), and baseline CRP concentrations (low *vs*. high) at study enrollment. Then, the association between CRP concentrations and life prognosis was statistically examined using a hazard ratio according to the Cox hazard proportional model.

The study was approved by the Bioethics Committee of National Hospital of Utano (approval no. 26–4). The Bioethics committee waived the need for informed consent due to the retrospective nature of the study and anonymity of the collected data.

### Patients

Consecutive patients with PD, who were treated at the Department of Neurology, the National Regional Center for Neurological Disorders and National Hospital of Utano from March 2004 to November 2007, were enrolled, and the medical records were retrospectively reviewed. PD diagnosis was based on the United Kingdom Parkinson’s disease Brain Bank Diagnostic Criteria. The enrollment criteria included PD patients free of any infection and who underwent measurements of plasma CRP.

### Chronological stability of baseline CRP

To investigate the tendency for “regression to mean,” plasma CRP was repeatedly measured when the patients were free of any infection. Patients were assigned into three groups according to plasma CRP levels upon study enrollment (low, middle, and high), and follow-up CRP concentrations were measured 4 times at most (21–180 days, 181–360 days, 361–720 days, and 721–1,080 days), when patients were free of any infections for 28 days prior to and after blood sampling. Follow-up CRP concentrations were separately investigated according to the bottom, middle, and top thirds of baseline CRP concentrations to investigate the stability of baseline CRP concentrations. The definition of “free of infection” included no use of antibiotics, no fever (body temperature > 37.5°C), and no findings of pneumonia upon chest X-ray.

### Survival time analysis

In survival time analysis, the observation period represented the time from study enrollment to the endpoint (date of death or May 16, 2014). Patients were censored when they were transferred to another hospital, and it was uncertain whether they were alive or dead. The study enrollment was the date of the first blood sampling for CRP measurements, which was performed for a routine blood test. Because of the possible association between CRP level baseline features, such as age, sex, PD duration and severity, cognitive function (Mini-Mental State Examination (MMSE), and nutritional condition (serum albumin level), we investigated the association using two methods, multi-variable analysis and stratifying analysis, according to clinical factors. Concerning the former, the association of baseline CRP levels was estimated using the hazard ratio (HR) according to the Cox proportional hazard model, which was adjusted for age, sex, mH-Y, PD duration, MMSE score, and serum albumin concentrations. In PD, the most common cause of death is pneumonia [15], and suffocation, fracture due to falls, dehydration, and unexpected sudden death are often seen in PD [16]; these causes can be considered to be related to PD. However, in contrast, vascular deaths or cancer deaths are not related to PD. Nevertheless, we examined the associations for all deaths. In addition, the associations with CRP and death, except for PD-unrelated events, were examined, and deaths from PD-unrelated events were censored or labeled “alternative outcomes.”

The following factors were collected for prediction variables; age, sex, PD duration, modified Hoehn-Yahr stages (mH-Y), MMSE scores, serum albumin, and plasma CRP concentrations upon study enrollment. In patients with motor fluctuations, mH-Y was evaluated in “ON period.” Although age and PD duration increased, and mH-Y and MMSE worsened during the observation period, all predictable variables were collected upon study enrollment, because the purpose of the study was to determine the associations of clinical features at enrollment and life prognosis.

The use of non-steroidal anti-inflammatory drugs (NSAIDs) at study enrollment was also collected in the current analysis (no use, current use, or habitual use at enrollment), because ibuprofen can be associated with PD risk reduction [17, 18].

### Cross-sectional association of CRP concentrations and PD progression

In addition to survival time analysis, the cross-sectional association between CRP concentrations and PD progression was assessed using a case-control study with data obtained upon study enrollment. After assigning patients who presented with rapid progression as “cases” and others as “controls,” the association was investigated as an odds ratio using a multivariate logistic regression model.

### Variables

Age, MMSE, PD duration (years), albumin (mg/dL), and CRP (mg/L) were regarded as scale variables. Due to the non-Gaussian distribution, log_2_ CRP [2], instead of CRP, was used in multivariable statistic models. Sex (male/female) was regarded as categorical, and mH-Y stage was handled as dichotomous (mH-Y 1–3 *vs*. 4–5) in the statistical analysis.

### Sample size and statistical analysis

Based on a previous study [19], the cumulative survival rate was assumed to be 80% and 60% for the lower two thirds and the top third of CRP concentrations upon study enrollment, respectively. The sample size was estimated at 219 with 90% power and *p* < 0.05.

Kaplan-Meier curves for the cumulative incidence of death were obtained after dividing patients into two groups according to clinical features. The log-rank test was used to determine the associations between life prognosis and clinical factors. The HRs of baseline CRP concentrations for deaths were estimated using the Cox proportional hazard model after adjusting for age (per 10 years), sex (male or female), PD duration (per 5 years), mH-Y (1–3 or 4–5), serum albumin (per mg/dL), and MMSE (> 24 or ≤ 24). By changing the cut-off value of the baseline CRP concentration, HRs were estimated with 95% confidence intervals. After stratifying patients according to age, sex, PD duration, mH-Y, MMSE, and serum albumin, Kaplan-Meier curves were used to confirm that the association between CRP and life prognosis was independent from these factors. Statistical significance was tested as a pooled *p*-value of a Log-rank test. *P*-values < 0.05 were considered statistically significant. All statistical analyses were performed using the statistical software IBM SPSS version 21 and Graph Pad software 5.0.

## Results

**Fig 1:**
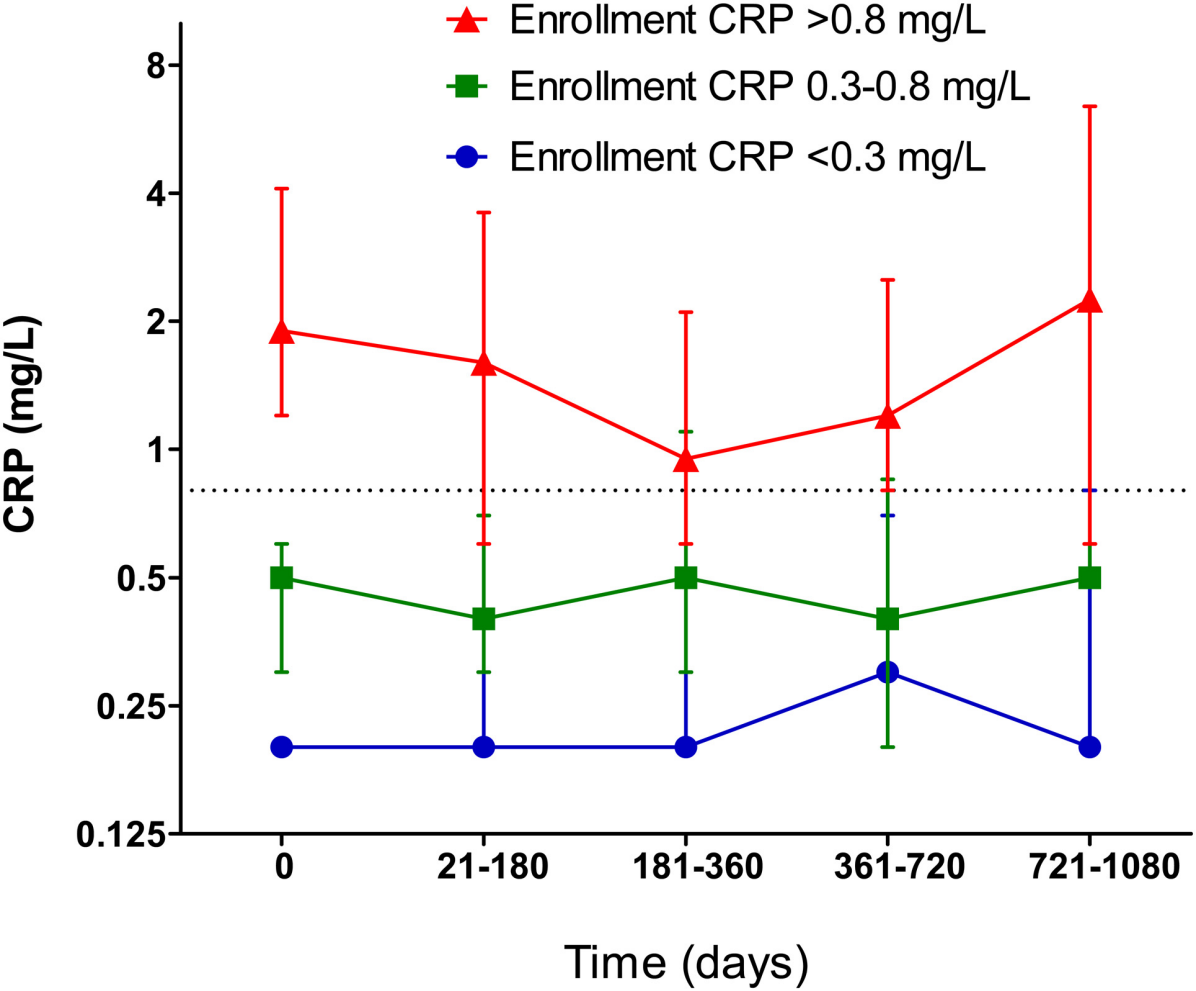
Serial changes in CRP levels in patients with low, mid, and high baseline CRP concentrations. CRP was measured at study enrollment and during the follow-up period (21–180 days, 181–360 days, 361–720 days, and 721–1080 days after study enrollment). Patients were assigned to three groups: those with low-level (< 0.3 mg/L), mid-level (0.3–0.8 mg/L), and high-level (> 0.8 mg/L) CRP at enrollment. Data represent median, and top and bottom error bars represent 75 and 25 percentile values, respectively.

The CRP concentrations at follow-up remained within the range of ≤ 0.8 mg/L in 77.5%, 75.5%, 75.8%, and 74.1% of patients with CRP ≤ 0.8 mg/L at the first sampling, as well as at the second (21–180 days), third (181–360 days), fourth (361–720 days), and fifth (721–1,080 days) follow-up blood sampling, respectively. Levels remained > 0.8 mg/L in 59.2%, 73.4%, 60.9%, and 56.1% of patients with CRP > 0.8 mg/L at the second, third, fourth, and fifth follow-up blood samplings, respectively. Fig 1 illustrates the chronological changes in baseline CRP concentrations, showing the stability of baseline CRP. Upon study enrollment, the mean CRP concentration was 1.53 mg/L, and after logarithm transformation, the mean log_2_ CRP was −0.79 ± 1.79 (± SD).

S1 Table summarizes the clinical features of the study patients. Age, mH-Y, and CRP were significantly higher, albumin was lower, and PD duration was longer in those who died compared with those who were still alive at study completion. S1 Fig is a histogram of log_2_ CRP measured upon study enrollment. The proportion of deceased participants to survivals increased with increasing log_2_ CRP concentrations.

The main causes of deaths were pneumonia (n = 23, 40.4%), sudden death (n = 11, 19.3%), cancer (n = 6, 10.5%), and suffocation (n = 3, 5.3%). Pneumonia is a well-known cause of death in PD and is associated with swallowing disturbances. In addition to pneumonia, suffocation is thought to be related to PD. In this study, sudden death was likely due to PD-related autonomic failure. One patient committed suicide due to PD-related depression. Another patient drowned in a river while in a state of confusion due to PD psychosis. Refractory hypoglycemia was identified in two patients with no history of insulin use or diabetes mellitus. The patients who died of hypoglycemia were emaciated and no cause of emaciation other than PD was identified. Therefore, death from refractory hypoglycemia was regarded as PD-related death. In contrast, cancer deaths, vascular deaths (stroke and pulmonary thrombosis), heart failure, and hepatic failure were regarded as PD-unrelated deaths.

**Fig 2:**
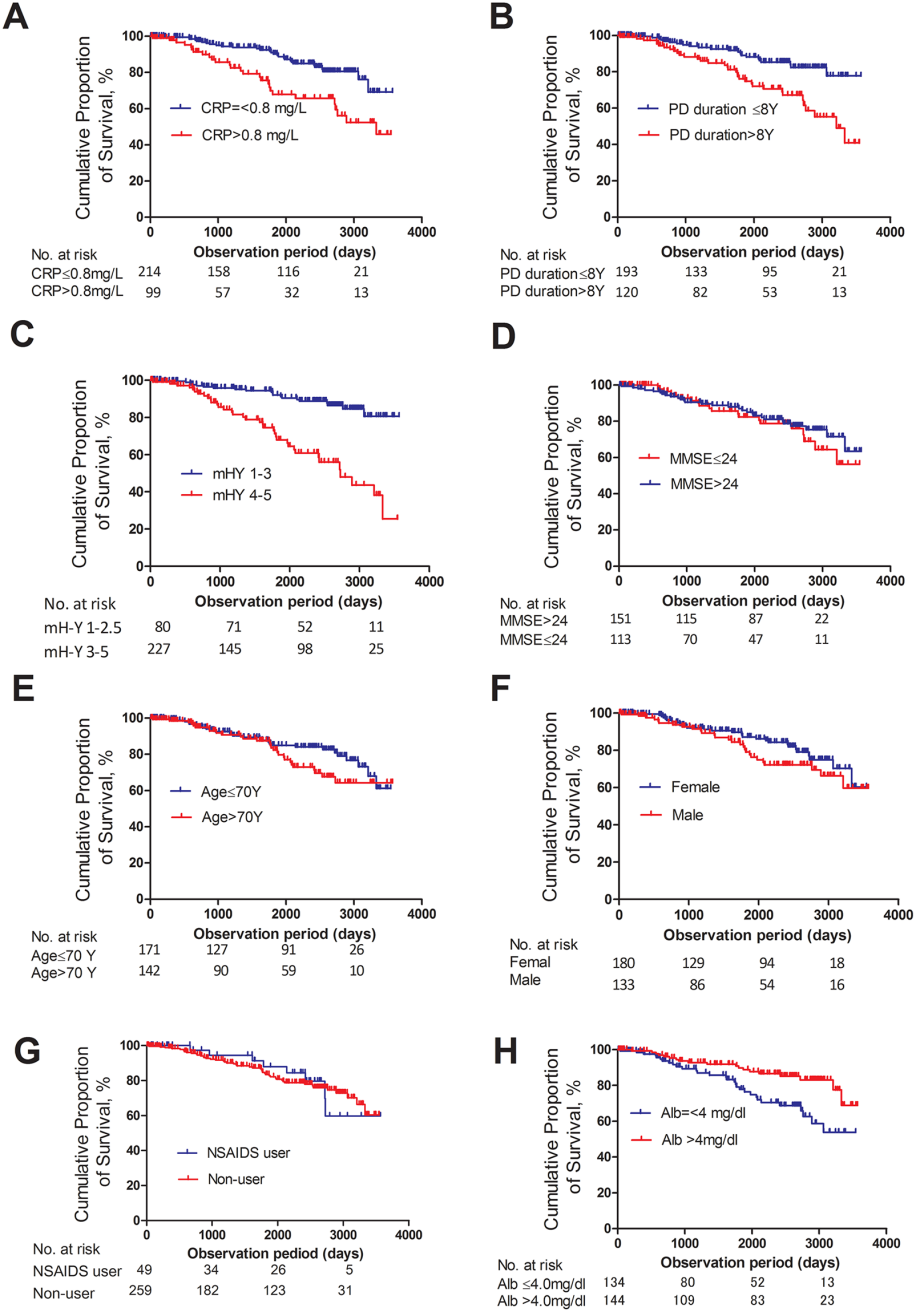
Survival curves of PD patients according to baseline clinical features. Cumulative survival rates according to CRP levels (A), PD disease duration (B), modified H-Y stages (C), MMSE score (D), age (E), sex (F), NSAID use (G), and serum albumin (H). There was a statistically significant difference in the survival rate between patients with CRP > 0.8 mg/L and ≤ 0.8 mg/L (*p* = 0.00004) (A), patients with PD disease duration ≤ 8 years and > 8 years (*p* = 0.00004) (B), those with modified H-Y 1–3 and modified H-Y 4–5 (*p* = 0.00001) (C), and those with albumin > 4.0mg/dL and albumin ≤ 4.0mg/dL (*p* = 0.004). However, the survival rate was not influenced by MMSE score, (*p* = 0.499) (D), age (*p* = 0.117) (E), sex (*p* = 0.186) (F), or NSAID use (*p* = 0.846). Statistical significance was tested using the Log-rank test.

S2 Fig provides scattered plots of the scale predictable variables. The plots showed no multicollinearity among age, PD duration, MMSE, serum albumin, and log_2_ CRP, although there was a slight but significant difference in CRP between mH-Y stages (S3 Fig). We generated Kaplan-Meier survival curves to compare life prognosis among the clinical factors. As shown in Fig 2, the cumulative survival rate was better in patients with CRP ≤ 0.8 mg/L than in those with CRP > 0.8 mg/L. Furthermore, the survival rate was significant higher in patients with PD duration ≤ 8 years compared with > 8 years, as well as in those with mH-Y 1–3 compared with mH-Y 4–5, and in those with albumin > 4.0mg/dL compared with ≤ 4.0mg/dL. In contrast, the survival rate was not significantly influenced by MMSE, age, or sex. There was no difference in survival time between NSAID non-users and current or habitual users [2,978 (2,836–3,119) days in non-users and 3,019 (2,698–3,341) days in current or habitual users].

**Fig 3:**
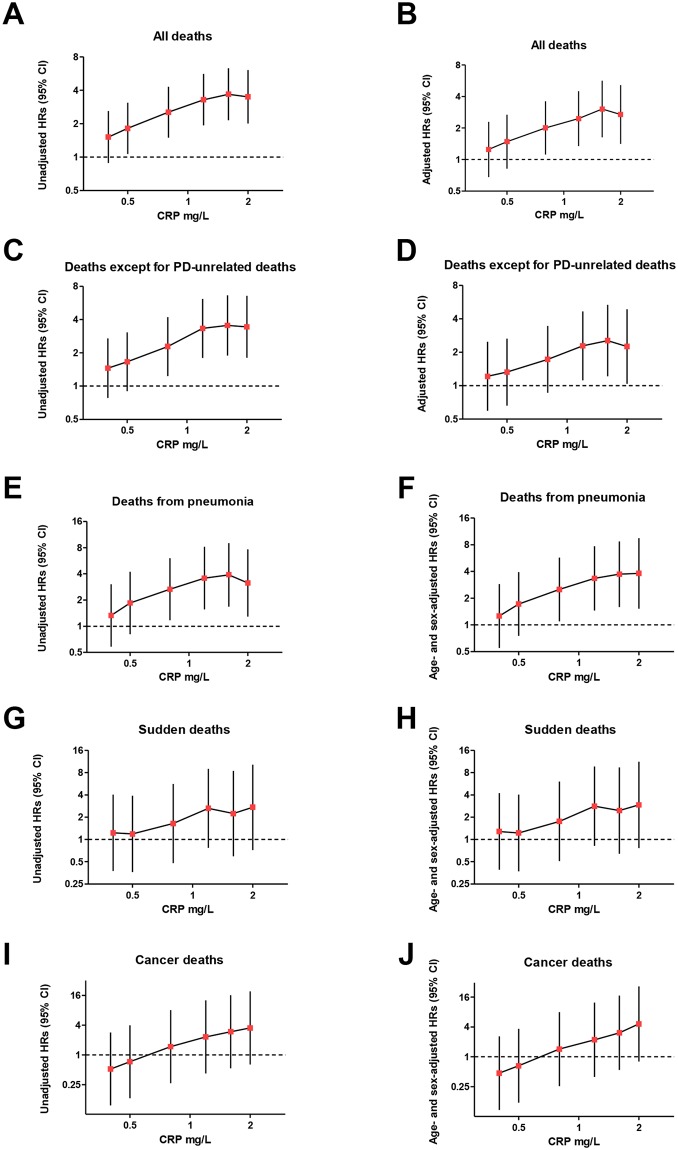
Relative risk of death by CRP concentration. Relative risk of death was estimated as hazard ratios (HRs) using Cox proportional hazard models. Unadjusted HRs of all deaths correlated with log CRP concentrations (A). To exclude possible confounders, HRs of all deaths were adjusted for age, sex, PD disease duration, MMSE (≤24 *vs*. >24), mH-Y (1–3 *vs*. 4–5), and serum albumin levels, and the analysis showed a log-linear association with CRP concentrations (B). Similarly unadjusted and adjusted HRs of death, excluding PD-unrelated deaths, correlated with CRP concentrations (C, D). Unadjusted and age- and sex-adjusted HRs of deaths from pneumonia correlated with CRP concentrations (E, F). Unadjusted and age- and sex-adjusted HRs of sudden deaths correlated with CRP concentrations, although there was no significance (G, H). Unadjusted and age- and sex-adjusted HRs of deaths from cancer correlated with CRP concentration, although there was no significance (I, J).

We also calculated the HRs for all deaths according to CRP level using the Cox proportional hazard model. The assumption for proportionality of hazards was met in log minus log plots (data not shown). Unadjusted HRs for all deaths increased with log CRP concentrations. The HRs adjusted for age, sex, PD duration, mH-Y, MMSE, and albumin for all deaths increased with increases in CRP concentration, and the associations between CRP and HRs were almost linear. After regarding PD-unrelated deaths (cancer deaths, vascular deaths, heart failure, and hepatic failure), as well as deaths from unknown causes, as censored due to “alternative outcomes,” the associations were very similar; the unadjusted and adjusted HR increased with log CRP concentrations. The HR values of deaths from pneumonia, sudden deaths, and cancer deaths were also almost linearly associated with CRP (Fig 3). The HR values of log_2_ CRP for all deaths and PD-related deaths are shown in Table 1.

**Table 1:** Relative risk of death by clinical features in Parkinson disease.

		All deaths		Deaths except for PD-unrelated events*	
baseline features		Adjusted Risk (95% CI)	*p*	Adjusted Risk (95% CI)	*p*
Age	*per* 10 years	1.79 (1.14–2.81)	0.012	1.40 (0.84–2.33)	0.197
Sex	Male	1.47 (0.80–2.72)	0.216	1.69 (0.82–3.50)	0.154
	Female	1 (ref)		1 (ref)	
PD duration	*per* 5 years	1.42 (1.09–1.86)	0.010	1.42 (1.03–1.95)	0.032
mH—Y	4–5	2.85 (1.40–5.80)	0.004	3.15 (1.34–7.38)	0.008
	1–3	1 (ref)		1 (ref)	
MMSE	≤24	1.99 (1.02–3.89)	0.044	1.98 (0.88–4.44)	0.097
	>24	1 (ref)		1 (ref)	
CRP	*per* two-fold	1.29 (1.10–1.52)	0.002	1.23 (1.01–1.49)	0.041
Albumin	*per* mg/dL	0.63 (0.29–1.37)	0.242	0.44 (0.17–1.12)	0.086

CI, confidence interval; mH-Y, modified Hoehn-Yahr stage; MMSE, mini-mental state examination; CRP, C-reactive protein

Adjusted risk was estimated by a Cox proportional hazard model.

* Death from PD-unrelated events: vascular deaths, cancer deaths, hepatic failure, and deaths from unknown causes.

**Fig 4:**
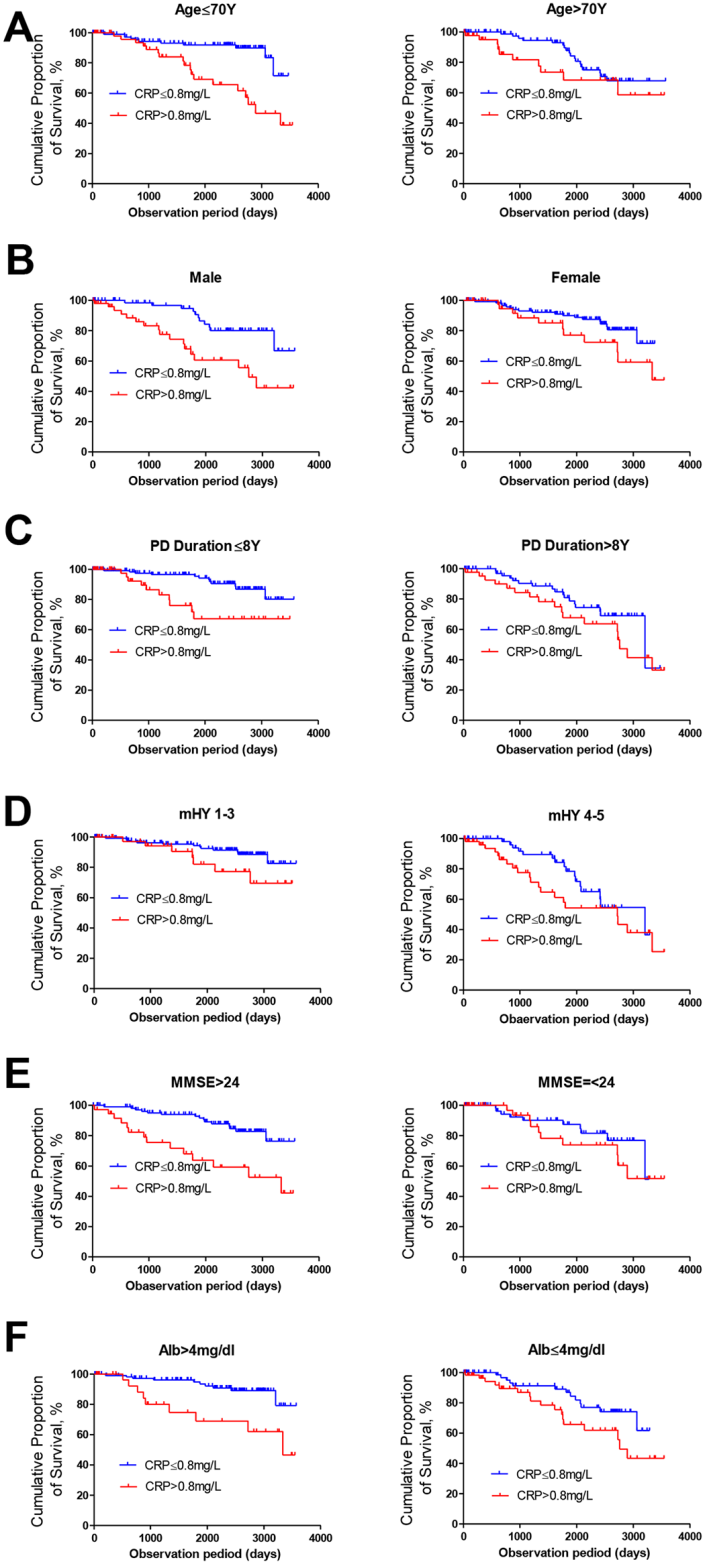
Survival curves of PD patients according to CRP levels. The cumulative survival rate was compared in patients with CRP ≤ 0.8 mg/L and those with CRP > 0.8 mg/L and was stratified by age (A), sex (B), PD duration (C), modified H-Y stage (D), MMSE (E), and serum albumin (F). The cumulative survival rate was significantly higher in patients with CRP ≤ 0.8 mg/L than those with CRP > 0.8 mg/L. Statistical significance was calculated in pooled models.

After stratifying patients by age (≤ 70 *vs*. > 70 years), sex (males *vs*. females), PD duration (≤ 8 *vs*. > 8 years), mH-Y stage (1–3 *vs*. 4–5), MMSE (> 24 *vs*. ≤ 24), and albumin (> 4.0 mg/dL *vs*. ≤ 4.0mg/dL), the Kaplan-Meier survival curves for CRP concentration (≤ 0.8 *vs*. > 0.8 mg/L) showed significantly higher survival rates for patients with CRP ≤ 0.8 mg/L compared with those with CRP > 0.8 mg/L (*p* < 0.05, Fig 4).

Finally, to determine the association of CRP concentration with PD disease progression, we compared CRP concentrations between cases with rapid progression and those with non-rapid progression using data obtained upon study enrollment. Rapid progression was defined as patients with mH-Y of 4–5 and PD duration ≤ 8 years. Multivariate logistic regression analysis that incorporated log_2_ CRP, age, sex, and MMSE (≤ 24 *vs*. 24) showed that log_2_ CRP and age were significantly associated with rapid progression (odds ratio; 1.34 (95% CI 1.12–1.60) *per* two-fold CRP concentration and 2.11 (95% CI 1.36–3.29) *per* 10 years of age). Another analysis in which rapid progression was defined as patients with mH-Y 4–5 and PD duration ≤ 5 years, identified log_2_ CRP, but not age, as a significant determinant of rapid progression (odds ratio; 1.36 (95% CI 1.05–1.75)).

## Discussion

The median CRP level in the present study was 0.50 mg/L, with a mean log_2_ CRP of -0.79, i.e., 0.58 mg/L (2^−0.79^ = 0.58 mg/L). These values are identical to those reported in our previous cross-sectional study of PD patients [20] and to those reported in a large population-based Japanese study [21], although they are lower than those reported in previous studies from other countries [2]. In contrast to the mean and median values, the standard deviation of log_2_ CRP (1.79) was similar to that reported in a previous meta-analysis study [2], suggesting a left-shift in the distribution in Japanese compared to other races. The reason for the low CRP in Japanese is unknown at present, but it might be due to genetic background rather than environmental factors, because CRP concentrations in Japanese Americans are also lower than other populations [22].

Our results showed poor life prognosis for PD patients with high baseline CRP values, long disease duration, high mH-Y stages, and lower serum albumin. While no multicollinearity was noted in predictable variables, we could not exclude possible confounders, because most patients with long disease duration also have advanced mH-Y stage and their CRP may be elevated. To exclude possible confounders, we confirmed the associations using Cox proportional hazard models; the obtained adjusted HR was linearly associated with CRP levels, as shown in Fig 3B.

Focusing on deaths from PD-related events, Fig 3B and 3D show the log-linear association of CRP levels with mortality after adjustment for age, sex, disease duration, mH-Y, MMSE, and serum albumin, indicating that the association is independent of age, disease duration, PD severity, cognitive function, and nutritional conditions.

Multivariate analysis by Cox proportional hazard model identified CRP levels, as well as age, mH-Y, and MMSE, as significant determinants of risk of all deaths and deaths from PD-related events (Table 1). These results are consistent with previous studies, which reported the association of PD mortality with severe H-Y stages [15] and poor cognitive function [23, 24].

In the general population, high CRP levels correlate with risk of vascular death [2] and cancer death [3], as well as the development of diabetes mellitus [22]. In this context, CRP was associated with mortality independent of PD. To resolve this issue, we computed the HR values of deaths from PD-related events after censoring patients who died of cancer, vascular events, and other PD-unrelated events. The analysis showed a significant correlation between CRP levels and PD-related deaths, and this correlation is independent of age, sex, disease duration, PD severity, and cognitive function. As shown in Fig 3I and 3J, the HRs of cancer death increased with increasing CRP. However, the HRs are not significant in this cohort, possibly due to the small number of cancer deaths. Two patients died of refractory hypoglycemia and were free of diabetes with no history of insulin use, but they were emaciated. While refractory hypoglycemia can be induced by certain drugs, no such drugs were prescribed. A similar case was previously reported and we assume that emaciation due to PD was associated with hypoglycemia in that case [25]. Although sudden death was categorized as PD-related death, we could not determine whether it was due to coronary insufficiency or PD-related autonomic failure. Although this is one limitation of our study, the high CRP concentrations were associated with mortality from other PD-related events, such as pneumonia. In addition, high CRP levels were also associated with rapid progression of PD. Taken together, these results support the conclusion that CRP levels correlate with prognosis of PD.

As shown in Fig 2, the use of NSAIDs upon study enrollment was not associated with life prognosis in PD, although medications can change during the long-term clinical course and may influence CRP levels or inflammation.

Previous studies have demonstrated neuroinflammation, such as microglia activation [10] and infiltration of CD4 lymphocyte [26], in the brains of PD patients. In PD, dying neurons are thought to release alpha-synuclein, and this in return is taken up by surrounding neurons where it accumulates in Lewy bodies of degenerating neurons [27, 28]. Alpha-synuclein is oxidized and nitrated, thereby inducing neuroinflammation [29, 30], and this latter process is probably mediated by Fcγ receptors [31], which bind to CRP [32]. CRP also increases permeability of the blood brain barrier through Fcγ receptors, further modulating neuroinflammation [33]. It is known that free radicals, including OH and NO, are generated as a byproduct of mitochondrial oxidative phosphorylation during ATP synthesis. In the presence of iron (Fe), these free radicals participate in the Fenton reaction to synthesize peroxynitrite ions involved in α-synuclein nitration and neuroinflammation [34–38]. Hence in addition to CRP, the ratio of nitrated α-synuclein/native α-synuclein may be used as an early and sensitive biomarker of neuro-inflammation in PD [38] (S4 Fig).

High CRP levels increase permeability of the blood brain barrier by binding to the Fcγ receptor [33, 39] and elicit microglia activation in the brain [32]. Animal studies have shown that systemic inflammation causes neurodegeneration through microglia activation [40, 41]. CRP is synthesized in the liver and is then transported to accumulate, or is synthesized in neurons, in neurodegenerative lesions in the brains of Alzheimer’s disease patients [8]. Recent evidence suggests that systemic inflammation contributes to the exacerbation of acute symptoms of chronic neurodegenerative disease [20, 42, 43], suggesting that systemic inflammation seems to promote neurodegeneration. Neuroinflammation can also elicit activation of inflammasome and caspase-1 in the brains of patients with Alzheimer disease [44]. Similar to Alzheimer disease, it is thought that aggregation of alpha-synuclein released from neurons can elicit neuroinflammation by microglia activation and inflammasomes in PD [45]. The present results add support to this hypothesis.

## Supporting Information

S1 Fig Histograms of log_2_ CRP. Distribution of log_2_ CRP was bell-shaped, and the proportion of patients who died during the follow-up (green) to those who are still alive (blue) increased with log_2_ CRP.
(TIF)

S2 Fig Scatter plots of scale predictable variables (age, PD disease duration, MMSE, and log_2_ CRP). There was no multicollinearity between these parameters.
(TIF)

S3 Fig Relationship between plasma CRP levels and PD severity (mH-Y). Plasma CRP levels were expressed by box-lots according to mH-Y stages. There was a statistically significant difference in CRP between mH-Y stages (one-way ANOVA, *p* < 0.0001).
(TIF)

S4 Fig Possible mechanism of subclinical systemic inflammation and neurodegeneration in PD. Subclinical systemic inflammation leads to an increase proinflammatory state, which elicits neuroinflammation and nitration of a-synuclein. This further causes microglia activation, inflammasome formation, and mitochondrial damage.
(TIF)

S1 Table Baseline clinical features by life outcomes in Parkinson disease. (PDF)
