# Supplementary figures and images for: Baseline C-Reactive Protein Levels and Life Prognosis in Parkinson Disease
Source: PLoS One. 2015 Jul 28;10(7):e0134118. doi: 10.1371/journal.pone.0134118 (PMC4517917; doi:10.1371/journal.pone.0134118)

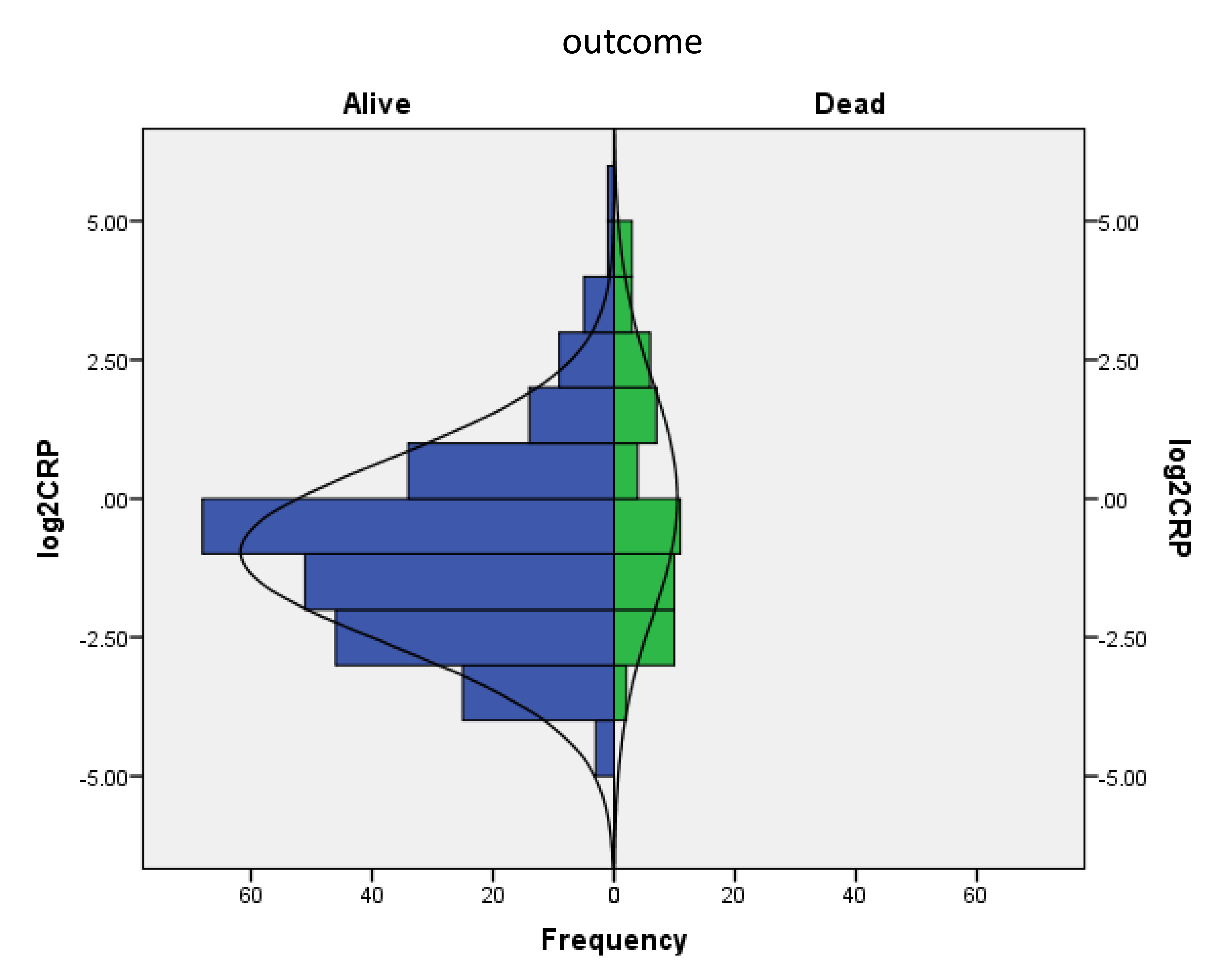

Supplement: S1 Fig — Distribution of log2 CRP was bell-shaped, and the proportion of patients who died during the follow-up (green) to those who are still alive (blue) increased with log2 CRP. (TIF) [file pone.0134118.s001.tif]

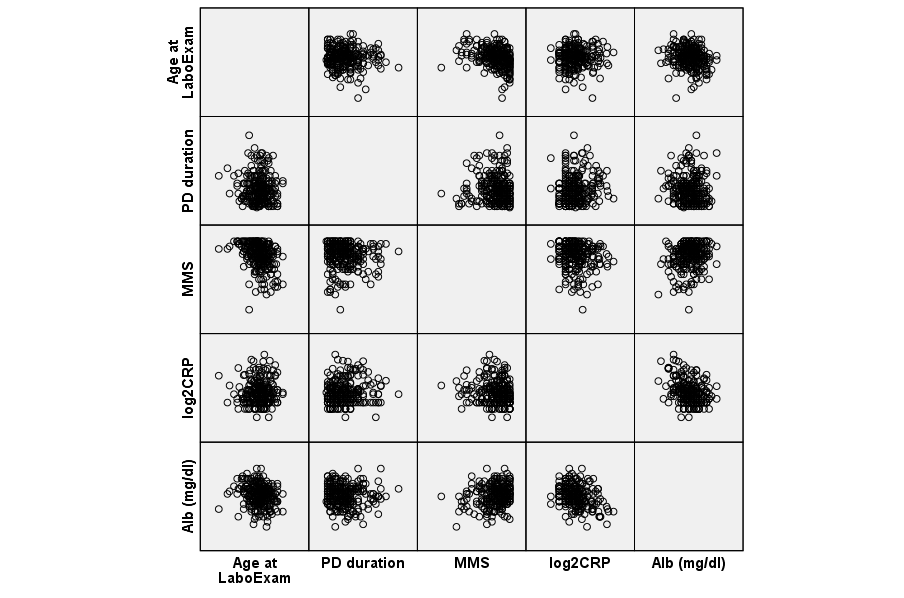

Supplement: S2 Fig — There was no multicollinearity between these parameters. (TIF) [file pone.0134118.s002.tif]

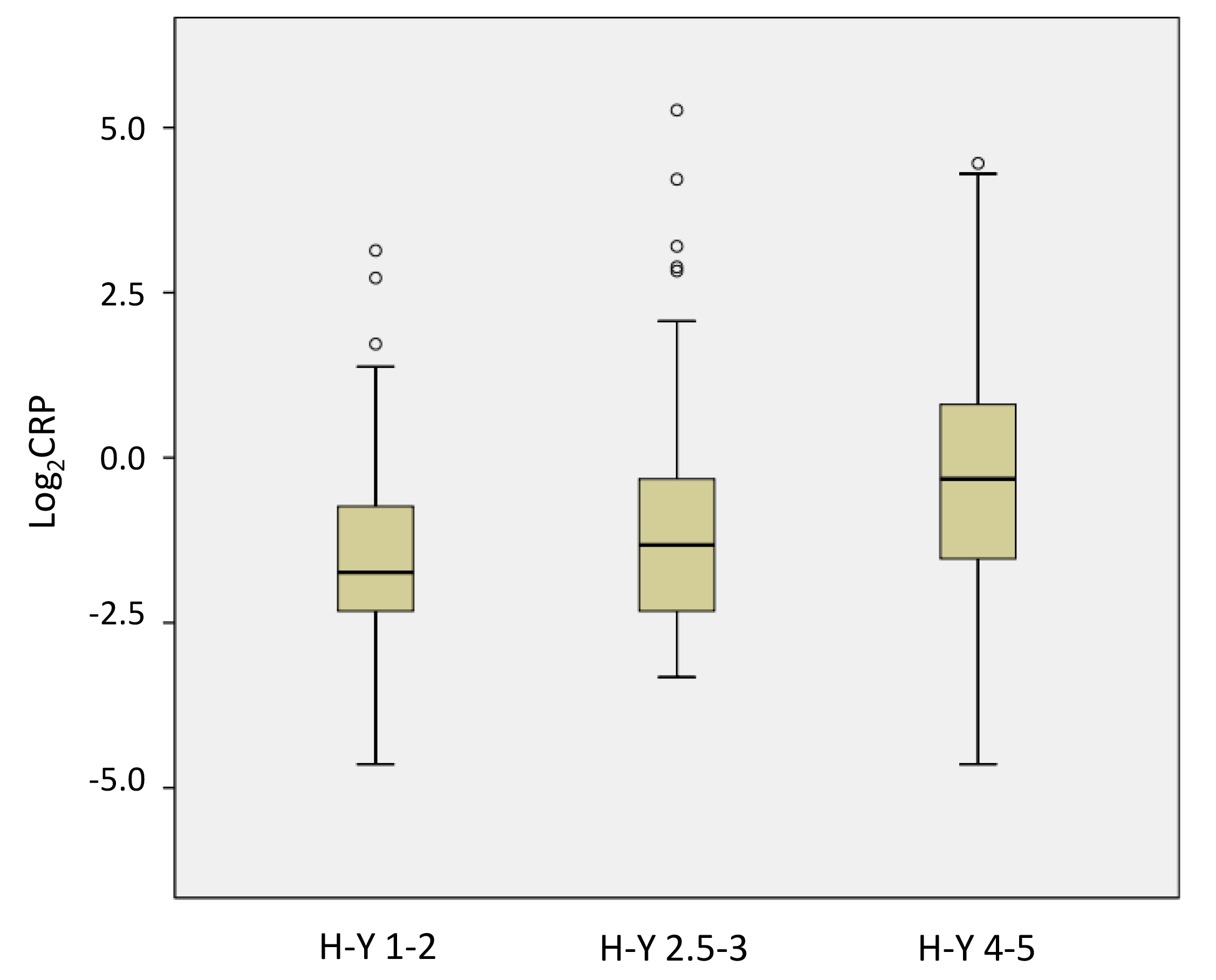

Supplement: S3 Fig — Plasma CRP levels were expressed by box-lots according to mH-Y stages. There was a statistically significant difference in CRP between mH-Y stages (one-way ANOVA, p < 0.0001). (TIF) [file pone.0134118.s003.tif]

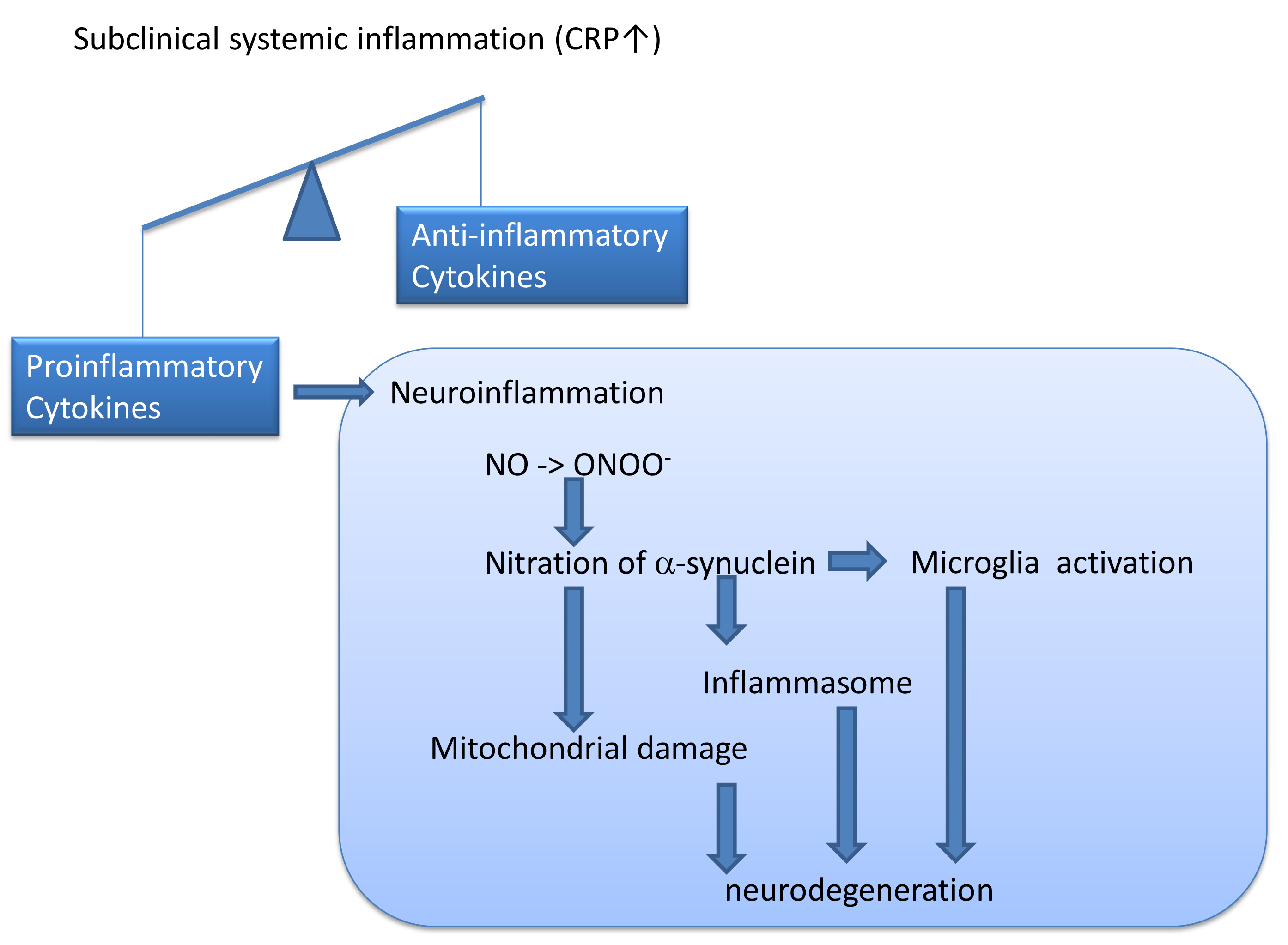

Supplement: S4 Fig — Subclinical systemic inflammation leads to an increase proinflammatory state, which elicits neuroinflammation and nitration of a-synuclein. This further causes microglia activation, inflammasome formation, and mitochondrial damage. (TIF) [file pone.0134118.s004.tif]
