# Supplementary material for: Baseline C-Reactive Protein Levels and Life Prognosis in Parkinson Disease
Source: PLoS One. 2015 Jul 28;10(7):e0134118. doi: 10.1371/journal.pone.0134118 (PMC4517917; doi:10.1371/journal.pone.0134118)
Supplement: S1 Table — (PDF) [file pone.0134118.s005.pdf]

S1 Table Baseline clinical features by life outcomes in Parkinson disease

		Dead (n=56)		Alive (n=257)		*p*
age (Y), mean (SD)		71.5	(7.7)	68.6	(9.5)	0.036 [1]
Sex, n (%)	Male	27	(48%)	106	(41%)	0.37 [2]
	Female	29	(52%)	151	(59%)	
mH--Y, n (%)	1-2.5	4	(7%)	76	(30%)	<0.00001 [2]
	3	16	(29%)	92	(37%)	
	4-5	36	(64%)	83	(33%)	
PD duration (Y), mean (SD)		10.6	(6.1)	7.3	(5.3)	0.00004 [2]
MMSE, mean (SD)		24.8	(3.8)	24.3	(5.3)	0.38 [2]
CRP (mg/L), median		0.8		0.5		0.014 [3]
Albumin (mg/dL), mean (SD)		3.9	(0.4)	4.1	(0.4)	0.030 [1]
NSAIDs	Non-user	47	(85%)	212	(84%)	0.840 [2]
	Current user	2	(4%)	7	(3%)	
	Habitual user	6	(11%)	34	(13%)	

SD, standard deviation; CRP, C-reactive protein

1, t-test; 2, Chi-square test; 3: ANOVA
